# A Survey of the Performance-Limiting Factors of a 2-Dimensional RSS Fingerprinting-Based Indoor Wireless Localization System

**DOI:** 10.3390/s23052545

**Published:** 2023-02-24

**Authors:** Abdulmalik Shehu Yaro, Filip Maly, Pavel Prazak

**Affiliations:** 1 Department of Informatics and Quantitative Methods, Faculty of Informatics and Management, University of Hradec Kralove, 500 03 Hradec Kralove, Czech Republic; 2 Department of Electronics and Telecommunications Engineering, Ahmadu Bello University, Zaria 810106, Nigeria

**Keywords:** indoor localization, RSS, fingerprinting, ML algorithm

## Abstract

A receive signal strength (RSS) fingerprinting-based indoor wireless localization system (I-WLS) uses a localization machine learning (ML) algorithm to estimate the location of an indoor user using RSS measurements as the position-dependent signal parameter (PDSP). There are two stages in the system’s localization process: the offline phase and the online phase. The offline phase starts with the collection and generation of RSS measurement vectors from radio frequency (RF) signals received at fixed reference locations, followed by the construction of an RSS radio map. In the online phase, the instantaneous location of an indoor user is found by searching the RSS-based radio map for a reference location whose RSS measurement vector corresponds to the user’s instantaneously acquired RSS measurements. The performance of the system depends on a number of factors that are present in both the online and offline stages of the localization process. This survey identifies these factors and examines how they impact the overall performance of the 2-dimensional (2-D) RSS fingerprinting-based I-WLS. The effects of these factors are discussed, as well as previous researchers’ suggestions for minimizing or mitigating them and future research trends in RSS fingerprinting-based I-WLS.

## 1. Introduction

Indoor wireless localization system (I-WLS) is a type of wireless positioning system (WPS) that is used to determine the location of a radio frequency (RF) transmitter situated within an indoor environment such as buildings and office spaces using a position-dependent signal parameter (PDSP) derived from the received RF signal with a localization algorithm [1,2,3]. The type of I-WLS depends on the type of wireless technology used, such as Wi-Fi, Bluetooth, Zigbee, and ultra-wideband (UWB) [4,5,6,7], the PDSP, and the type of localization algorithms, which are fingerprinting, angulation, and lateration. According to [1,5], time of flight (TOF), direction of arrival (DOA), receive signal strength (RSS), round trip time (RTT), and channel state information (CSI) are the most commonly used PDSPs for an I-WLS. The TOF of an RF signal is the time taken by the transmitted RF signal from the transmitter to be detected at the antenna of the receiver. The RF transmitter and the receivers must be in perfect synchronization to achieve good localization accuracy [1]. The DOA is the arrival angle of the RF signal observed at the receiver. It does not require synchronization between transmitter and receiver; however, it requires an antenna array to be used at each receiver for DOA estimation, making it more expensive and consuming more power [5]. The CSI is a collection of information that describes how an RF signal propagates from the RF transmitter to the receiver [8,9]. This information not only includes attenuation but also the scattering and refraction encountered by the signal during its propagation [10]. CSI-based I-WLS has a very high localization accuracy of less than 1 m and better stability than the RSS. CSI, on the other hand, is not widely available in Wi-Fi enabled devices, unlike RSS, because it requires advanced network interface cards, which are not commonly found in today’s smartphones [11]. Furthermore, as the size of the database increases, so do the training costs and processing complexity, and CSI information can only be obtained via a Wi-Fi signal [9,11]. The RTT is the time that it takes for an RF signal to travel from the transmitter to the receiver and back. It does not require synchronization, as the TOF does; however, device manufacturers mainly decide to disable RTT measurements by means of software [12]. The RSS is the average received power of an RF signal detected at a receiver, and it is assumed to follow an exponential decay model, which is a function of path loss and distance between the RF transmitter and receiver [1]. RSS has an advantage over TOF as it does not require perfect synchronization between the RF transmitter and receiver; it does not require an antenna array as in the case of DOA-based systems; and it can be obtained from any RF signal, unlike the CSI, which can only be obtained from a Wi-Fi signal. Compared to RTT, RSS is simpler, more cost-effective, more scalable, and more accurate in environments with high signal interference. The TOF and DOA rely on physical distances, that is, the range between the transmitter and the receiver [11]. As such, line-of-sight (LOS) is always assumed. As for the RSS, only the lateration-based localization algorithm relies on physical distance. The fingerprinting-based localization algorithm relies on RSS measurements mapped to the reference locations from which they are obtained. Due to the complex nature of indoor environments owing to the presence of walls, furniture, and humans obstructing the path between the RF transmitter and receiver [3,11], the RSS is the most effective PDSP for the indoor localization and will be the focus of this survey. Table 1 shows a comparison of the different PDSPs used for indoor localization.

As previously stated, the RSS-based I-WSL uses either fingerprinting or lateration algorithms as its localization algorithm [13,14]. The lateration algorithm is a range-based method that uses the physical distance between the RF transmitter and receiver for localization. It uses the log-normal path loss model, which relates the RSS measurement at each receiver to the physical distance between the transmitter and receiver. The lateration algorithm is a well-established algorithm for RSS-based indoor localization, but non-line-of-sight (NLOS) effects, multipath issues, the need for specialized antennae, and precise synchronization requirements make it less effective for practical application [11]. The fingerprinting algorithm is widely used for RSS-based I-WLS due to its availability, affordability, and lack of additional infrastructure requirements; it can be used with a variety of wireless technologies [4,11,15,16,17]. As such, it will be the focus of this survey. In the fingerprinting algorithm, the RSS values of the received RF signals from wireless access points (APs) are collected at different reference locations using an RSS indicator (RSSI) receiver. The RSS values and the reference locations from which they are obtained are used to construct an RSS-location database known as a radio map or fingerprint database [8]. Table 2 shows the acronyms and abbreviations used throughout the paper.

The remainder of the paper is organized as follows: Section 2 summarizes some earlier published surveys on 2-D RSS fingerprinting for indoor wireless localization. In Section 3, a brief discussion on the localization process of the RSS fingerprinting base I-WLS is presented, and the factors limiting the performance of the system are presented in Section 4. A brief discussion on the RSS datasets available online is presented in Section 5, which is followed by discussions on RSS fingerprinting system performance metrics in Section 6. The conclusion and future research trends are in Section 7.

## 2. Existing Survey on 2-D RSS Fingerprinting-Based I-WLS

There are several survey papers on fingerprinting-based I-WLS [5,8,15,18,19,20,21,22,23,24,25], and this section outlines the survey papers on RSS fingerprinting-based I-WLS to differentiate our survey from existing surveys. A summary and comparison of the contributions of each of the survey papers is presented in Table 3.

An overview of the different localization systems, techniques, and wireless technologies used by the I-WLS is presented in [5]. The focus of the survey is on the comparison of the different indoor localization systems in terms of cost, localization accuracy, advantages, and disadvantages. The survey also elaborated on the different localization algorithms used by the DOA, TOF, and RSS-based systems. The survey fails to discuss issues limiting the performance of the system and how these issues can be overcome. Challenges associated with the implementation of an I-WLS were identified and presented [8]. The discussions focused on challenges associated with RSS measurement acquisition and the design of machine learning algorithms to handle variations in RSS measurements. Other important issues such as the choice of wireless technology and its effect on system performance as well as RSS radio map generation were not presented. In [21], an overview of the use of smartphone sensors with various indoor localization techniques is presented. The survey focuses on how smartphone sensor data can be used with various indoor localization techniques. Another survey on smartphones based on I-WLS using Wi-Fi and Bluetooth as RF sources is presented in [18]. The survey focuses on problems associated with the practical implementation of the system, which include RSS fluctuations, device heterogeneity, radio map generation, and localization time. However, the effects of these problems—for instance, the density of the radio map affects the localization time, and RSS fluctuation affects the reliability of RSS measurement, which subsequently affects the localization accuracy—and the techniques to overcome the challenges imposed by these issues were not presented. Another survey on indoor localization systems is presented in [22], and their discussions focused on evaluating the various systems in terms of energy efficiency, availability, cost, reception range, and latency. Factors affecting the performance of the system such as RSS measurement reliability, radio map generation, and choice of RF signal source were not discussed. An overview of the different Wi-Fi-based I-WLS, divided into active and passive localization systems, is presented in [25]. Comparative analysis based on accuracy, cost, scalability, and reliability as well as system deployment challenges were presented, which included multipath effects, NLOS propagations, and device heterogeneity. Challenges such as radio map generation and other factors that could cause fluctuations in Wi-Fi signals were not discussed.

Few survey papers focused their discussions on fingerprint-based I-WLS [4,6,15,24,26]. In [4], the discussion focused on fingerprinting-based machine learning techniques used in indoor localization. The survey also fails to discuss issues limiting the performance of the system. A survey on fingerprinting-based localization systems that rely on Bluetooth is presented in [6]. The focus of the survey is on the stages of the Bluetooth-based fingerprint localization system and the classification of the methods used in the different stages. There were no discussions on issues limiting the performance accuracy associated with each stage. A survey on localization issues related to radio map generation, outlier detection, and device heterogeneity for a fingerprinting-based I-WLS is presented in [15]. However, other localization-related issues such as the ML algorithm and the effect of temporal and ambient conditions were not presented. Deep learning methods for fingerprinting-based I-WLS are extensively discussed in [16]. However, discussions were not focused on localization performance-limiting factors such as radio map generation and RSS measurement reliability.

During the preliminary stage of an I-WLS deployment, it is important to recognize potential issues that may arise post-deployment. These issues, how they affect the system after deployment, and possible solutions to mitigate the effects of these issues must all be known. The density of the radio map, for example, is an issue that must be addressed. The higher the density of the radio map, the higher the localization accuracy; however, this comes at the cost of the localization time. If a system for real-time localization is to be deployed, the possible solutions to this problem must be known. Most of the previously mentioned surveys [4,5,8,15,18,21,22,23,25] focused their discussions on either a comparison of different localization technologies and techniques using various performance metrics or briefly introduced some of the issues affecting the system’s localization performance without discussing how these issues affect the system’s localization performance or suggesting possible solutions to these issues. Furthermore, most surveys on 2-D RSS fingerprinting-based I-WLS [6,15,16], which is the scope of this current survey, only focused their discussions on issues such as RSS outliers, device heterogeneity, radio map detection, and ML algorithms, which are not the only issues that affect the localization performance of the RSS fingerprinting-based system. Besides that, the impact and potential solutions to mitigate the impact of the issues were not presented. As a result, this survey identified and presented as many factors as possible that impact the system’s 2-D localization performance. These factors will be divided into two categories: online phase factors and offline phase factors. The impact of these factors on system localization performance is discussed, and a summary of current solutions proposed in previous research works is presented.

## 3. RSS Fingerprinting-Based 2-D Localization Methodology

The localization process of the fingerprinting-based I-WLS using RSS as PDSP is described in this section of the survey paper. 

**Figure 1:**
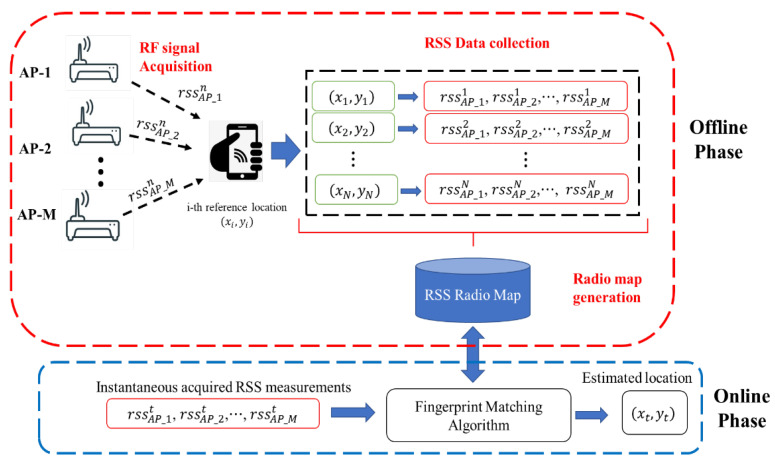
Fingerprinting-based indoor localization process.

The RSS fingerprinting-based localization process is in two stages, namely the offline phase and the online phase, as shown in Figure 1 [8]. The offline phase first involves the reception of RF signals from the wireless APs using a smartphone at reference locations. This is followed by the determination of the RSS values of each of the received RF signals and finally the construction of a radio map, which is a database of RSS vectors mapped to the coordinates at which they are obtained.

Let there be M wireless APs, and $rss_{AP\_M_{\;}\;}^{n}$ be the RSS value of the signal transmitted by the *m*-th AP obtained at the *n*-th reference location with coordinates $\left(x_{n},y_{n}\right)$. The RSS measurement vector obtained from M APs at the *n*-th reference location is given as [1,6]:

(1) $$RSS^{n}=\left[rss_{AP\_1_{\;}\;}^{n},rss_{AP\_2}^{n},\cdots ,rss_{AP\_M}^{n}\right]$$

For N reference locations, the RSS measurement vectors based on Equation (1) for each location are obtained. These are mapped to the reference location where they are obtained and saved in an online database [8]. The online database is known as a radio map.

The second stage, which is referred to as the "online phase", involves the real-time localization of an indoor user by matching the instantaneously acquired and generated RSS measurement vector by the user with the RSS measurement vectors earlier stored in the radio map using ML algorithms [4,8]. For instance, let $RSS^{t}$ in Equation (2) be the instantaneously acquired RSS measurement vector by a user at an unknown location.

(2) $$RSS^{t}=\left[rss_{AP\_1}^{t},rss_{AP\_1}^{t},\cdots ,rss_{AP\_M}^{t}\right]$$

Given $RSS^{t}$, the ML algorithm searches through the radio map to find a reference location that has the same RSS measurement vector. That is, if

(3) $$RSS^{t}≅\;RSS^{n}\to \;\mathrm{for}\;1\leq n\leq N$$

then the location from which it is obtained is assumed to be the instantaneous location of the indoor user, that is:

(4) $$\left(x_{n},y_{n}\right)\to \;\left(x_{t},y_{t}\right)$$

Several research works have reported on different fingerprinting ML algorithms, but the most commonly used are K-nearest neighbor (KNN) and its different variances [8,27,28], support vector machines (SVMs) [5], random forest [4,29], and Gaussian mixture models [30].

Each phase of the localization process has factors that affect the overall performance of the system. Some of these factors directly affect the localization accuracy of the system, while others affect it in other ways. In the next section, the system performance limiting factors in the offline phase and online phase are presented with their effects on system performance and possible solutions suggested by previous research.

## 4. Performance Limiting Factors for RSS Fingerprint-Based I-WLS

The factors that affect the RSS fingerprinting-based I-WLS’s performance are categorized as offline phase factors and online phase factors and are presented in this section. First, the offline phase factors and their effects are discussed, followed by the offline phase factors and their effects.

### 4.1. Offline Phase Performance Limiting Factors

The offline phase, being the first phase of the localization process, is the overall system performance determining phase [31,32]. This is because the ML algorithm used in the online phase uses data—that is, RSS measurements obtained from the offline phase—for real-time localization of the user. However good an ML algorithm is, its localization accuracy will depend on the accuracy of the input data from the offline phase [32]. As was previously mentioned in Section 3, the offline phase involves three main activities: choosing a wireless technology, acquiring RSS measurements, and generating radio maps. The challenges that are related to each of the aforementioned activities and their effects on the system’s overall performance will be explored.

#### 4.1.1. Choosing a Wireless Technology

The selection of an RF signal source for use by the I-WLS is critical because it determines the deployment cost, system coverage, and susceptibility to environmental factors such as multipath and non-line-of-sight (NLOS) propagation. Researchers [4–6, 18] have identified a number of wireless technologies from which RSS measurements can be obtained. For the RSS fingerprinting-based I-WLS, Wi-Fi Technology, Bluetooth, Zigbee, and ultra-wideband are the most frequently used RF sources. A brief discussion of each of these technologies is presented as follows:

- Wi-Fi Technology

Wi-Fi networks are commonly used in various indoor environments such as offices, university campuses, homes, hotels, shopping malls, and hospitals, which makes them best suited for the localization of indoor users [4]. It operates within RF bands of 2.4 GHz for the IEEE 802.11b/ba/bb/bc/bd/be/g/n standard and 5 GHz for IEEE 802.11a/ac/ad/af/ah/ax [5]. The majority of portable devices, including laptops and smartphones, presently support Wi-Fi technology. Two location parameters, RSS and CSI, can be derived from the Wi-Fi signal. Wi-Fi-based I-WLSs have a number of advantages, which include rapid deployment, flexibility, and low installation cost [4,5,6,18]. Wi-Fi reception range has been demonstrated to be about 100 m, but according to recent research, it has increased to about 1 km [5]. Wi-Fi’s dependence on the density of reference locations for localization accuracy—the more reference locations there are on the radio map, the better the accuracy—is a significant drawback [4]. The size of the radio map grows with a greater number of reference locations, but this also lengthens the time it takes to locate a user. Deep learning techniques such as convolutional neural networks (CNN), artificial neural networks (ANN), deep belief networks (DBN), auto-encoders (AE), and recurrent neural networks (RNN) have been used in a few studies to shorten localization times [33].

- Bluetooth

Another RF source used for indoor localization is Bluetooth. It is based on the IEEE 802.15.1 standard [5] and uses the same ISM 2.4 GHz frequency band as the Wi-Fi IEEE 802.11 b/g standard [34]. It operates in the 2400–2483.5 MHz frequency range. Cost-effectiveness, extremely low power consumption, low transmission power, faster connection speed, high transmission rate, secure and effective communications, and accessible solutions are just a few of the benefits of using Bluetooth for indoor localization [4,5,6]. Additionally, it has a localization accuracy of about 4 m and a high scan rate of about 1 sec, which is faster than Wi-Fi with a scan interval of about 4 sec [18]. It is also resistant to sporadic outliers brought on by interference or the multipath effect [34]. Bluetooth 4.2, also referred to as Bluetooth low energy (BLE), is an upgraded version of traditional Bluetooth with an increased reception range of about 100 m in line-of-sight (LOS) conditions [5]. Furthermore, the recently released Bluetooth 5.2 has an improved connection range up to 200 meters (m) in line-of-sight (LOS), but under specific circumstances, such as in an open area, the range can be up to 400 m.

- Ultra-wideband

One of the emerging RF sources used in an I-WLS is UWB. An UWB signal has a carrier frequency greater than 2.5 GHz and an absolute bandwidth greater than 500 MHz [5]. Ultra-wide band signals have several properties, including low power consumption, less interference, effective penetration through walls and dense materials, and less sensitivity to multipath due to their short pulse duration, making them a good candidate for use in an I-WLS [4, 5]. Compared to Wi-Fi and BLE, it has been reported that an RSS-based UWB I-WLS has a higher localization accuracy of about 0.5 m [18]. Smartphones with UWB chips such as Apple’s (iPhone 11, 12, 13, and 14) and Samsung’s (Galaxy Note 20 Ultra) are very expansive, which is one of the limiting factors affecting their wide range of application scenarios [35].

- Zigbee

Another RF source used for RSS-based indoor localization is Zigbee. It is based on the IEEE 802.15.4 wireless standard and operates on the 2.4 GHz, 900 MHz, and 868 MHz frequency bands. Compared to the Wi-Fi standard, Zigbee has a lower deployment cost, a lower data transfer rate, and a shorter latency time with a localization accuracy of about 5 m [36]. Zigbee has some drawbacks, such as a limited positioning range, poor anti-interference performance, and a greater multipath effect [4]. Additionally, not all mobile devices are equipped with Zigbee technology.

Several research papers [4,5,6,18,19,37] have reported on system implementation performance comparisons with different wireless technologies. Table 4 shows a comparison of the differences between RSS-based wireless technologies considering localization accuracy, power consumption, and deployment range. The advantages and disadvantages of each wireless technology are also presented.

Each wireless technology has advantages and disadvantages, as shown in Table 4. Wi-Fi and Zigbee-based I-WLS are the best when it comes to deployment over a large indoor environment because they have LOS reception ranges up to 100 m. However, both systems have moderate localization accuracy and are extremely susceptible to changes in the environment, human error, and the multipath effect. When compared to Wi-Fi and Zigbee-based systems, Bluetooth-based I-WLS has better localization accuracy and uses less power because it is primarily battery-operated, but its deployment range is very limited. The UWB-based I-WLS has the best localization accuracy with low power consumption and is extremely vulnerable to the multipath effect, but it is more expensive than Wi-Fi, Zigbee, and Bluetooth-based systems.

**Figure 2:**
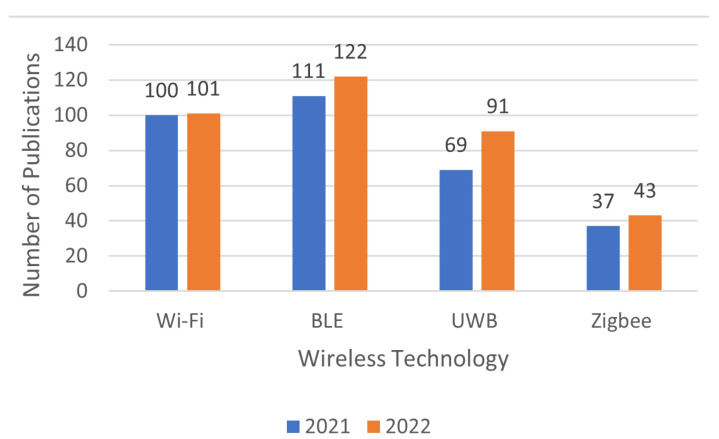
RSS fingerprinting-based I-WLS publication frequency trend for different wireless technology.

Shang et al., (2022) presented an analysis of research records from 2011 to 2020 in the Web of Science database to demonstrate the level of research interest in the various wireless technologies used for I-WSL using techniques such as keyword search [4]. Analysis of the data showed that between 2011 and 2020, Wi-Fi as an RF source for the I-WLS was the most popular. In this paper, the analysis is extended to the years 2021–2022, and the results for the publication frequency trend are shown in Figure 2.

According to the data gathered, BLE-based research is higher in 2021 and 2022 which is followed by Wi-Fi and UWB when compared to research conducted in previous years but in total, Wi-Fi-based researches are much higher that BLE-based research. The current increase in the BLE-based research is due to its low-power consumption when compared with Wi-Fi and most of the current localization devices are battery operated. However, in terms of localization accuracy, the Wi-Fi-based systems have a better localization accuracy [4,6,8]. 

The UWB is not currently the most popular technology, but there is a significant increase in its research between 2021 and 2022 when compared to other technologies. UWB has higher localization accuracy, lower latency, and greater robustness when compared to other technologies, particularly in harsh environments and under NLOS conditions. In recent years, smartphone manufacturers such as Apple and Samsung have equipped their devices with UWB chips to take full advantage of high-precision localization and tracking with air tags and Galaxy smart tag devices, respectively. UWB technology is expected to be used in future smartphone-based I-WLS deployments [38,39].

#### 4.1.2. Acquiring RSS Measurements

The accuracy of the system’s localization is directly correlated with the accuracy of the RSS measurements. As earlier mentioned, the accuracy at which the RSS measurements are obtained determines the accuracy of the localization algorithm in the online phase. Due to the device configuration, hardware variations, multipath effect, and NLOS, RSS measurements for various measuring devices at any given reference location differ. Additionally, it changes with the time of day that the measurement is made as well as with various indoor environmental factors. In summary, the factors listed below contribute to RSS measurement fluctuation:

- Ambient conditions

The ambient conditions refer to the nature of the indoor environment at the point where RSS measurements were taken for the generation of a radio map. Opening and closing doors and windows, the presence or absence of a crowd, the presence or absence of furniture in an indoor environment, and the number of pieces of furniture present are all examples of this [8]. If the ambient condition at which the radio map was created is different from the condition at which instantaneous localization takes place, then the accuracy of the system is greatly affected. An analysis of the effect of ambient conditions on the reliability of RSS measurement is presented in [40]. Using a BLE signal source at the same reference location with the same transmitter and receiver power, it was found that there was a difference of about 6 dBm in the RSS measurement when taken in the presence of humans and without humans. As such, to ensure better localization accuracy, the ambient conditions during the generation of the radio map in the offline phase and localization during the online phase should be approximately the same.

- Temporal conditions

Temporal conditions include factors such as environmental temperature and the time of day at which RSS measurements were taken for radio map generation. These factors greatly affect RSS measurement reliability. For instance, RSS measurements taken during the winter will differ from RSS measurements taken during the summer. So also, RSS measurements taken during the day differ from RSS measurements taken during the night. In [41], an experimental analysis was carried out to determine the variation of the RSS measurement with the time of the day it was taken. Using a smartphone, LTE RSS was measured at a fixed reference location between the hours of 7:30 a.m. and 11:00 p.m. at 30 mins intervals for a period of 30 days. The result of the experiment shows that the RSS measurement varies with the time and with the day on which it is taken.

- Device heterogeneity

Because of differences in device configuration and hardware, RSS measurement will differ between two RSS measuring devices. Device heterogeneity becomes an issue if the devices (APs and RSSI receivers) used for the generation of the radio map in the offline phase are different from the devices used in the online phase localization process. As a result of differences in hardware, RSS measurements obtained using a Samsung smartphone will be different from those obtained using an iPhone. Due to device configuration differences, RSS measurements obtained with different devices of the same brand will also differ. The use of different APs during the offline phase and online phase will also result in RSS measurement variation due to differences in AP hardware configurations such as transmit power or operating frequency. Several studies [42,43] examined the effect of device heterogeneity on RSS measurement and concluded that different devices result in different RSS measurements. Solutions to deal with device heterogeneity have been proposed, which include the linear transformation method (manually adjusting RSS values for each distinct testing device) [44], the use of collaborative mapping (generation of a linear mapping function through training online measured RSS values using unsupervised machine learning algorithms) [45], and the use of an alternative location fingerprint such as signal strength difference (SSD) instead of absolute RSS values [46]. As for variation in RSS measurement due to differences in APs, it is important to ensure that the configuration of the APs used during the generation of the radio map is the same during the localization process. However, if a variation in AP configurations is noticed, a new RSS radio map is to be built using the new configuration.

- Advertising on different carrier frequency

The carrier frequency used in the transmission of the RF signal also contributed to the fluctuation of the RSS measurements. Variations in the RF carrier signal during radio map generation and online localization degrade the localization performance of the system. The BLE is linked to the transmission carrier frequency difference issue. The BLE has three advertisement channels, namely channels 37, 38 and 39, each with center frequencies of 2.402, 2.246 and 2.48 GHz. Modern smartphones usually switch between the three advertising channels to transmit BLE beacons, which results in RSS fluctuations. It was reported that the difference in RSS values acquired at the same reference location on different advertisement channels was found to be between 6 dB and 15 dB [40,47]. Thus, it is important to identify the advertising channel used by the BLE transmitter during the radio map generation in the offline phase. It is difficult to know the channel on which a BLE beacon was transmitted, as this information is often unclear to the driver at the receiver. In [47], an attempt to identify BLE beacon advertisement channels was made by studying the BLE scanning patterns on different smartphone models. It was observed that after every reset of the BLE device, the scanning starts from channel 37, and this could be tracked using an Android application. The most effective way to address the BLE advertising channel issue is to take a snapshot of RSS measurements over time and aggregate the RSS measurements [40,48].

- Orientation of RSS measuring device

The differences in the orientation of the RSS measuring device during radio map generation and the localization in the online phase result in variations in RSS measurement under the same experimental conditions. When RSS is obtained using devices at different orientations, there can be a difference of about 30 dB between the minimum and maximum RSS value at constant transmitter-receiver distances [40,49]. Thus, it is important to ensure that the same device orientation is maintained between the two phases of the I-WLS.

- Interference in ISM band

Many wireless systems use the ISM band; for instance, BLE and Wi-Fi use the 2.4 GHz band. RSS fluctuations can be caused by RF interference, especially when W-Fi and BLE modules coexist on the same chip and share the same antenna, as in most smartphones. Interference between these two technologies is well documented in the literature; for instance, a 6 dB fluctuation was observed using a BLE beacon at the same transmitter-receiver distance due to the multipath effect and interference from a Wi-Fi AP [50]. Another experiment was carried out by placing a BLE transmitter directly under a Wi-Fi AP, and a 75% drop in the RSS was observed between the Wi-Fi AP being turned on and off, equating to about a 10 dB drop in the RSS [51], whereas in [40] it was determined to be approximately 6.8 dB. Despite IEEE recommendations for ISM-band coexistence [52], interference mitigation techniques for BLE, such as a backoff strategy and an adapting frequency hopping pattern, were developed to avoid BLE transmission on WLAN spectrum [53].

To summarize, each of the aforementioned factors causes a change in RSS measurement at a given reference location under the same experimental conditions. Other research works propose techniques to deal with RSS measurement regardless of the factor contributing to the RSS fluctuation [29,48,54,55,56,57,58,59,60,61,62], in addition to the solutions proposed by previous research works on dealing with each of the factors mentioned above. A summary of these techniques can be seen in Table 5.

The most common technique for addressing RSS fluctuation, as shown in Table 5, involves acquiring multiple RSS measurements for each reference location and utilizing a smoothing technique, such as a Kalman filter or moving average, to obtain a mean representation of the RSS measurements.

The most common technique for addressing RSS fluctuation, as shown in Table 4, involves acquiring multiple RSS measurements for each reference location and utilizing a smoothing technique, such as a Kalman filter or moving average, to obtain a mean representation of the RSS measurements. 

#### 4.1.3. Radio Map Generation

The generation of the RSS database, also known as the radio map, is the last activity of the offline phase of the I-WLS localization process. The RSS database contains received RSS values from an RF signal and is mapped to the reference location at which they were obtained. Besides directly affecting the localization accuracy of the system, the approach taken to the generation of radio maps also determines the system set-up time, deployment cost, localization time, and system complexity. The conventional approach to the generation of radio maps is the site survey approach, which involves the collection of RSS values at known locations, which is time-consuming and labor-intensive [63,64,65]. To expedite radio map generation with reduced labor cost, several techniques have been proposed, with the most commonly used being the path loss propagation model [65], radio map interpolation [65], simultaneous localization and mapping (SLAM) [66], and active and passive crowd sourcing [67]. Each of these techniques is discussed in detail below:

- Path loss propagation model

This is a simulation-based approach to the generation of the RSS radio map. Using indoor wireless propagation models such as the ray tracing model, multiwall, and log-distance path loss models to deterministically estimate the RSS values at given reference locations from the fixed AP [68,69]. This approach drastically reduced the system deployment time; however, it has several disadvantages, such as deficient performance in complex indoor environments, the inability to build a precise model due to multipath effects, shadowing, and delay distortions.

- Radio map interpolation

Another technique to cut down on site survey time is the use of interpolation. It is known to be the easiest way to add missing or additional RSS fingerprints at certain reference locations [70]. RSS measurements are taken at some points using either the site survey method or the pathloss model, and the RSS of the remaining reference locations will be determined using interpolation. Several interpolation techniques have been used, some of which are the Kriging spatial interpolation algorithm [71], distance-based interpolation, and inverse distance weighted interpolation [65]. The use of interpolation comes with a lot of disadvantages, some of which are a decrease in performance with an increased number of APs and the inability to build an accurate model in the case of the wireless pathloss interpolation technique.

- Simultaneous localization and mapping

Simultaneous localization and mapping (SLAM) is the technology that enables a mobile robot equipped with sensors such as LiDAR, GNSS receiver/antenna, and an inertial measurement snit (IMS) to map an unknown environment and at the same time position itself based on the built map [72]. This technology plays a great role in the automatic generation of RSS radio maps while at the same time cutting labor costs. Radio map generation using SLAM is performed in two steps [67]. The indoor environment in which the system is to be deployed is first mapped by the robot using the SLAM algorithm. The second step involves the collection of RSS measurements from APs by driving the robot through the indoor environment. The obtained RSS measurements and their estimated locations are processed to generate the radio map. One of the major disadvantages of SLAM is that it has a heavy computational load for resource-limited smartphones.

- Active crowd sourcing

This involves the active participation of users working within an indoor environment in the generation of an RSS radio map [28,67,73]. Users are given a dedicated device, which they voluntarily use for measuring signal power. The dedicated device usually includes a map on which users mark the approximate locations at which they measure the signal power. The measured signal powers and their geotags, collected by each individual user, are pushed and stored in an online database for further processing. This method significantly reduced the time and labor costs associated with radio map generation, but it has a number of drawbacks, including intentional fraud from participants, wasted storage space on the radio map server due to the multiplicity of fingerprints from the same location labeled by different users, being prone to RSS fingerprint blind spots, and being highly sensitive to variations in devices capturing the measurements.

- Passive crowd sourcing

In contrast to the active crowdsourcing method, in which users manually measure signal power and geotag them, passive crowdsourcing measures signal power and geotags them using inbuilt device sensors such as IMS [74,75]. There is no human intervention in the generation of the radio map. Examples of IMS are an accelerometer, a magnetometer, a gyroscope, and a barometer. Some of the disadvantages of passive crowdsourcing methods are delays in map construction due to the unavailability of GPS signals, limited applicability due to hand-device implementation constraints, low accuracy, and the accumulation of IMS errors.

Each of the site survey alternative techniques to radio map generation has its advantages and disadvantages, as mentioned earlier. A summary of the limitations of these alternative techniques is shown in Table 6.

Table 7 presents summaries of a few current research studies on alternate site survey methods for radio map generation.

### 4.2. Online Phase Performance Limiting Factors

The second phase of the fingerprint-based I-WLS localization process, known as the "online phase," involves determining the instantaneous location of a user by matching the instantaneously obtained RSS measurements by the user with RSS measurements in the radio map using ML algorithms. Several ML algorithms have been proposed, and a brief introduction to some of the most commonly used ML algorithms is presented below.

- k-nearest neighbor algorithm

The k-nearest neighbor (k-NN) algorithm is the most widely used RSS-based ML algorithm due to its simplicity and estimation accuracy comparable to other ML algorithms. In the k-NN algorithm, an instantaneously obtained RSS vector from a user is individually compared with the RSS vectors in the radio map. The degree of matching between the two RSS vectors is characterized by the distance between the two vectors. The most commonly used distance measurement is Euclidean distance; however, other distance measurement techniques such as Manhattan distance, Minkowski distance, and Hamming distance can be used [78]. The obtained distance measurements between the user’s location RSS vector and all RSS vectors in the radio map are ranked in ascending order, and the average of the K number reference points with the smallest distance is taken as the actual user location [4]. One of the issues with the k-NN is poor data labeling during the averaging process. To improve the data labeling accuracy, the use of weighted distance was proposed [4,61,79,80]. The weighted modified version of k-NN is known as weighted k-NN (Wk-NN). The choice of "k" used by the k-NN or Wk-NN has a significant effect on the performance of the algorithm. The higher the value of "k," the higher the matching accuracy of the k-NN algorithm, but the higher the computational cost of the system [7]. To enable optimal selection of the "k" value, an adaptive k-NN algorithm was proposed [27,81]. The adaptive Wk-NN algorithm has the best matching accuracy compared to all the variants of the k-NN algorithm. Another technique to improve the k-NN algorithm, especially when dealing with large radio map sizes, is the use of clustering algorithms such as k-mean and c-mean [4]. This will considerably shorten the calculation time and improve the real-time performance of the k-NN-based I-WLS. The k-NN algorithm, despite being the most used ML algorithm for the I-WLS, has a lot of disadvantages, some of which include being sensitive to noisy and missing RSS fingerprints and not being able to perform well when dealing with large fingerprint datasets.

- Support vector machine

Another ML algorithm used in the online phase of the fingerprint-based I-WLS is the support vector machine (SVM). It was originally designed to solve binary classification problems but was further expanded to solve multi-class classification problems. In the case of linearly separable data, SVM uses hyperplanes to define decision boundaries for separating data points of different classes [82] and the kernel function for nonlinear classification [83]. The aim of SVM is to construct a hyperplane with the maximum margin between different classes. In most cases, data are not perfectly linearly separable, making the separating hyperplane susceptible to outliers; thus, a limited number of misclassifications should be tolerated around the margins. SVM is a fast and dependable algorithm that performs very well with a limited number of RSS fingerprints [83]. It has the advantage of fast convergence and is easy to construct. The localization performance of SVM is closely related to its parameters; thus, poorly selected parameters will lead to poor localization accuracy [4]. Several researchers, including [82,83,84], have used SVM as a fingerprint ML algorithm.

- Gaussian mixture model

Fingerprint-based localization can also be performed using conditional probability. For instance, during the offline phase, instead of using the RSS measurement average, the RSS measurement probability distribution is used [30,85]. The Bayesian inference concept, based on the estimated density function approximated as Gaussian, is used to estimate the location of the indoor user. This approach has a better handling of noise in wireless channels [85]. In practical applications, RSS measurement distributions are far from a Gaussian distribution, but the use of several Gaussians may give a better representation of these measurements [85]. The Gaussian mixture model (GMM) is a probabilistic approach that allows for the approximation of the probability density function of the RSS measurement from APs by a weighted sum of Gaussian components, with each component characterized by several parameters estimated iteratively using an expectation maximization (EM) algorithm [27,30].

- Random forest

Another fingerprint localization algorithm that has been shown to outperform SVM, k-NN, and GMM is the random forest algorithm, as this localization algorithm can provide high localization accuracy when dealing with large fingerprint datasets [4, 86, 87]. To enable accurate localization when dealing with a large fingerprint dataset, the entire dataset is divided into two sub-datasets, namely the training dataset and the testing dataset. The training dataset is used to train the random forest algorithm, while the test dataset is used for simulation tests to examine the performance of the random forest algorithm with different hyperparameter tuning. The trained random forest algorithm model that shows better performance is saved for use in the online localization phase. During the online localization phase, the obtained real-time RSS measurements are fed into the trained random forest algorithm to determine the indoor mobile user’s current location. This phase consumes less computation time compared to the offline phase [86].

- Deep learning

When dealing with a large RSS radio map dataset or a complex environment, the computational time of a conventional fingerprint ML algorithm increases, which increases the localization time. Researchers have attempted to apply deep learning-based feature extraction methods to indoor localization with the aim of reducing localization time and improving localization accuracy [16,33,48,78,88]. The RSS-based I-WLS using the deep learning-based feature extraction method first involves the pre-processing of RSS values before feeding them into the feature extraction method [4,33]. The pre-processing involves normalization, calibration, augmenting, and/or dimensionality reduction using statistical methods or traditional ML algorithms such as SVM. Hierarchical features of the RSS values are extracted using deep neural networks (DNN) such as the convolution neural network (CNN), the deep belief network (DBN), the recurrent neural network (RNN), and the artificial neural network (ANN) [4,33]. In the offline phase, the DNN uses the RSS values of all APs in the RSS radio map and the reference location as the training data to obtain the mapping relationship between the RSS and the reference location. During the online phase, instantaneously acquired RSS values for mapping relationships are inputted to predict the location. DNNs such as DBN and ANN are simpler with moderate performance accuracy. CNN has better localization accuracy, but it is more complex and demands higher computational resources [33]. Future I-WLSs will be based more on smartphones, using the aggregation of multiple sensors, but their computational ability is limited. Hence, a smartphone RSS-based I-WLS should be implemented, leveraging its multiple sensors with a simple neural network such as an ANN or a DBN. However, if localization is to be conducted online on a remote server, CNN is the best solution [48,88].

Table 8 provides a summary of the advantages and disadvantages of the ML localization algorithms which were previously discussed. The localization time and accuracy of the system are both impacted by the single activity that takes place during the online phase of the I-WLS-scanning the radio map to determine the user’s location. If accurate RSS measurements are obtained during the offline phase, then the localization accuracy will depend on how accurately the ML algorithm pinpoints a user’s location. There are numerous research findings on the localization performance and accuracy of different ML algorithms, but it is currently challenging to assess them using a single benchmark.

The size of the RSS radio map is one of the factors that directly impacts localization time, as was already mentioned. Localization times increase with RSS radio map size; however, a number of methods have been reported to help reduce RSS radio map size, some of which include dimensionality reduction and the use of clustering approaches [46]. The localization time of any ML algorithm is tied to its computational complexity (CC). The total amount of time needed for an algorithm to complete its execution is known as the CC. The lower the CC, the faster the execution. Table 9 compares the computational complexity of the most popular ML algorithms for I-WLSs using the big O notation [89,90].

A localization algorithm’s critical component, the CC, must be kept within acceptable bounds while maintaining high localization accuracy. If this is not taken into consideration, a system may have very high localization accuracy, but the improvement may not be compelling, meaning that instantaneous localization may be impossible. Since most localization algorithms require a sizable amount of storage and computational power, it is crucial to consider these factors when choosing a localization algorithm, particularly for devices with limited processing and storage capabilities, such as smartphones.

## 5. Online RSS Fingerprint Datasets

As previously stated, there is no unified RSS dataset to compare the localization accuracy of ML algorithms. There are, however, several RSS datasets available online that could be used to evaluate the performance of each localization ML algorithm [91,92,93,94,95]. Following is a brief discussion of some of these RSS datasets:

- UJIIndoorLoc Dataset

This is a well-known, publicly accessible multi-building, multi-floor indoor localization RSS database that is used to test I-WLSs ML algorithms that rely on WLAN or Wi-Fi fingerprints. The dataset was created in 2013 and made available to the public in 2014. It can be found in [91]. RSS data were collected from three adjacent multi-story buildings with at least four floors on the Jaume I University campus in Spain. In each room, reference locations were chosen to be in the middle and in front of the door(s) leading to the room. A total of 25 Android devices were carried by at least 20 different participants and used to collect about 520 RSS measurements from 520 APs scattered across the buildings at 933 reference locations. The RSS value of a detected AP ranged from 0 dBm (a very strong signal) to −104 dBm (a very weak signal). Undiscovered APs were assigned a value of −100 dBm. On average, 27 APs were detected per reference location. Five percent of the collected samples were set aside for testing.

- Dataset described in [95]

The dataset described by **[95]** was gathered over a 15-month period. The dataset’s primary goal was to provide researchers with the data they needed to study a system’s robustness against short- and long-term Wi-Fi signal variations. Short-term variations are caused by multipath and shadowing, while long-term variations are caused by changes in the environment and network. RSS Wi-Fi measurements were taken on two identical floors (3rd and 5th) of a 12 by 18 $m^{2}$ library wing with 106 reference locations per floor using a Samsung S3 smartphone. Consecutive observations facing the same directions were collected multiple times per month at each reference location. During a month, 15% of the samples collected were allocated for training, while the remaining 85% were allocated for testing, except for the first month’s samples, which were allocated for 73% training and 27% testing. In the last month of the sample collection, a total of 63,504 samples were collected. Each sample contained a timestamp, ground truth floor number, RP coordinates, and RSS values for all detected APs during the entire scan. In the first month, 77 APs were used, which gradually increased to 448 APs by the fifteenth month. The authors recently updated the dataset to include 40,080 new samples, corresponding to an additional 10-month collection period with 172 newly detected APs **[96]**. There are MATLAB scripts included that allow you to load a desired set based on filtering criteria.

- RSS dataset described in [94]

A dataset that includes approximately 14 h of annotated wearable measurements collected from four single- and two-story residential homes with four to eleven rooms was created by [94]. A custom-built, wrist-worn transmitter sent accelerometer measurements via BLE radio (in advertising mode) to several custom-built anchor nodes deployed throughout the residence. Each node records the RSS of the advertised packet and timestamps it upon receipt. Fiducial floor tags placed 1 m apart throughout the house provided ground truth location labels. A downward-facing camera strapped to a participant’s navel area automatically captured the floor tags as the participant walked across them. To account for the shadowing effect imposed by the participant’s body, data were collected at each floor tag facing each of the four cardinal directions. Furthermore, the dataset included samples generated from both scripted and unscripted scenarios. Scripted scenarios depicted participants walking quickly or slowly through the house, whereas unscripted scenarios depicted participants going about their daily lives. The dataset also includes annotated data collected from "activity zones," which are specific locations that correspond to specific activities such as cooking in the kitchen, eating at the dining table, or relaxing on the sofa. The dataset contains approximately 730,000 samples in total. Python scripts are provided for loading the dataset from the repository.

- RSS dataset described in [93]

A general-purpose dataset that can be used for positioning, tracking, proximity/occupancy detection, and social interaction detection was introduced by [93]. A 16.6 by 14.3 $m^{2}$ research facility with eight rooms, a connecting corridor, and 277 reference locations spaced 0.6 m apart was used for data collection. A Raspberry Pi with two BLE modules was placed in each room. While the other module broadcast advertisements at a frequency of 10 Hz, one module continuously searched for signals. Similar to this, mobile users were used to enable data collection both ways by carrying a smartphone that served as a receiver and a BLE tag that served as a transmitter. Each sample contains a timestamp, transmitter ID, receiver ID, and RSS value, totaling 2,820,000 samples in all. A separate file that maps timestamps to the coordinates of the RPs provides ground truth location data.

- SODIndoorLoc

A supplementary RSS dataset to meet with the demand not been meet by earlier published RSS dataset was developed by [92]. The supplementary open dataset for Wi-Fi indoor localization based on RSS covers three multi-floor buildings with a combine area of 8000 $m^{2}$. The 3 buildings have 105 pre-installed APs, of which 56 are single-band and 49 are dual-band. A total of 1802 points were placed in various locations; the number of reference locations was 1630, and the number of testing locations was 272. There are three scenes considered: the office room, the meeting room, and the corridor, from which a total of 23,925 samples are collected: 21,205 for training and 2,720 for testing. Reference locations for two buildings with one floor and a three-story building are 1.2 m and 0.5 m apart, respectively.

- RSS dataset described in [41]

This data was created using the RSS of cellular networks (LTE, GSM, and HSPA). Using a smartphone, cellular RSS measurements were taken around Covenant University, Nigeria, in an indoor scenario [41]. The RSS dataset was created to investigate the best network for mobile subscribers on roaming services and local subscribers’ high performance and data rates, which are also vital for indoor localization applications. Measurements were taken daily from 7:30 a.m. to 11:00 p.m. for 30 days, from July 1st to 30th July 2020. The primary device collected data at an interval of 30 min within the time range, while the supporting devices randomly collected data during the day.

The previously mentioned RSS datasets are available online for use in evaluating the localization performance of the ML algorithm. However, there are few RSS datasets that are online but not open for use by general researchers, some of which are **[97]**. The dataset was created using RSS information from more than 40 Wi-Fi APs collected over several days in an indoor environment. RSSI measurements were taken on the 4th floor of Building DJ, located at the East 9th Teaching Building of Huazhong University of Science and Technology, China. A total of six classrooms and a corridor, with a combined area of 717 $m^{2}$, were used. Classes were labeled using odd and even numbers, and the size of a class with an odd number is 10.5 × 9.56 $m^{2}$, and that of a class labeled with an even number is 10.5 ×7.76 $m^{2}$, while the corridor is 32.6 × 3.6 $m^{2}$. Table 10 shows a comparison of the characteristics of the earlier mentioned online RSS datasets.

## 6. RSS Fingerprinting-based I-WLS Performance Metrics 

Several parameters are used to evaluate the performance of the RSS fingerprinting-based I-WLS, and the most used parameters are discussed in this section.

- Localization accuracy

This is defined as the difference between the estimated user location and the actual or true user location. Most, if not all, wireless localization systems are designed to have very high localization accuracy of less than 0.5 m. However, there is also a trade-off between localization accuracy and other system performance metrics. For instance, the denser the RSS fingerprint database is, the higher the localization accuracy, but this comes at the cost of a longer localization time. Another trade-off is localization accuracy versus system complexity. Most complex localization algorithms have higher localization accuracy, but this usually comes at the expense of localization time and computation resources. The mean absolute error (MAE) and the root mean square error (RMSE) are the two commonly used parameters to measure the localization accuracy of the system. Mathematically, the MAE is obtained as follows [89]:

(5) $$MAE=\frac{1}{n}\overset{n}{\underset{i}{\sum }}\left|{\hat{y}}_{i}-y_{i}\right|$$

while the RMSE is obtained as [87]:

(6) $$RMSE=\sqrt{\frac{1}{n}\overset{n}{\underset{i=1}{\sum }}{\left|{\hat{y}}_{i}-y_{i}\right|}^{2}}\;\;\;$$

where ${\hat{y}}_{i}$ is the estimated location and $y_{i}$ is the true or actual location

- Complexity

The CC is the primary concern of any localization algorithm. It involves the time and resources required to carry out localization. Within the context of time, the CC of a localization algorithm is the time it takes to determine the location of an indoor user, while within the context of resources, the CC is the amount of storage or resources needed to carry out localization. A near-real-time localization process is one of the primary objectives of any wireless localization system. However, there is always a trade-off between localization time and accuracy. Table 8 presented earlier shows the time CC of some localization algorithms. The amount of storage space or resource a localization algorithm requires should also be taken into consideration when choosing a localization algorithm, and it usually depends on where the localization process will take place. In a situation where localization will take place on a resource-limited device such as a smartphone, the localization algorithm that requires limited resources should be used; however, when localization will be conducted in an online environment with unlimited resources, it is possible to use a localization algorithm that requires a large number of computational resources.

- Scalability

This refers to the ability of the system localization coverage area to be increase or accommodate the addition of new devices or users. If a system’s coverage area can be extended without any downtime in its usage such system is said to have an excellent scalability. Most I-WLS are design with the intension of increasing its localization area in future. There are several ways to increase the localization area of a system some of which are increasing the transmission power of the APs, and the use of interpolation techniques to extend the coverage area of an RSS radio map. So, when designing a system, it is good to ensure that room is given for future extending of localization area of the system.

- Cost

Another performance metric that is often overlooked is the cost of developing and deploying the RSS fingerprinting-based system. Cost is a function of the size of the localization area, the required localization accuracy, the technology used, power consumption, and a lot more. For instance, fingerprint construction using IMS only requires a smartphone and no additional infrastructure. In addition, some wireless technologies, such as Wi-Fi and BLE, require APs to be deployed, and most indoor environments are equipped with Wi-Fi APs for other applications that can be extended to localization. If a system requires no additional infrastructure or is based on existing infrastructure such as Wi-Fi or the use of IMS, then such a system is said to have a low deployment cost.

- Robustness

The ability of the system to withstand adverse conditions, such as component malfunction and signal losses, is referred to as robustness. Even if one or two infrastructure components fail or malfunction, the system should be able to perform performance localization. A Wi-Fi- or BLE-based system should function properly even if one or two Wi-Fi APs or BLE beacons fail to function.

## 7. Conclusions and Future Research Trends

Research on indoor localization based on fingerprinting is very active, and numerous efforts have been made to ensure the system’s higher localization accuracy in a variety of indoor environmental conditions. Several factors that are either in the online phase or offline phase of the localization process affect how well the I-WLS performs. This survey provided information on the various factors that either directly or indirectly influence the I-WLS’s performance. System performance limiting factors in the offline phase are thought to be the most important factors influencing overall system performance and should thus be given more consideration. For the online stage of the localization process, RSS data from the offline phase is required. Due to inaccurate RSS data, even the best localization ML algorithm cannot guarantee a precise localization estimate. The recommendations made by earlier research on how to minimize or eliminate the effects of these factors are presented.

Furthermore, three future research directions are proposed that, if explored, will significantly improve the performance of an RSS fingerprinting-based system. Future research should concentrate on the following topics:

- Periodic update of fingerprint database: It is a well-known fact that the characteristics of indoor environments change over time. Changes could include furniture placement or the construction of walls. As a result, the RSS database must be updated on a regular basis to reflect changes, which can be time-consuming and tedious. Future research should focus on developing a technique that can detect changes in the indoor environment and automatically re-calibrate the RSS database without the need for human intervention. A possible technique could be to use SLAM or smartphone sensors along with other DNN algorithms.
- Heterogenous RSS fingerprint database: The RSS fluctuation has a significant impact on the localization accuracy of the RSS fingerprint-based system. RSS fluctuation can be either short-term (due to the multipath effect, device orientation, and NLOS) or long-term (due to temporal-ambient conditions and device heterogeneity). Most RSS datasets are built with only one of these factors in mind. More experimental research in this area is required to enable the development of a heterogeneous RSS dataset that considers RSS fluctuations caused by both short-term and long-term factors. This will allow for the development and training of ML algorithms that are robust and capable of working in any indoor environmental condition without requiring significant modification.
- System performance trade-off: Another area that requires further investigation is the best trade-off between system performance metrics. The trade-off between localization accuracy and other performance measures such as scalability, complexity, cost, localization time, and power consumption is an example of this. Most systems are designed with high localization accuracy and, to some extent, low localization time in mind. This usually results in systems that are not scalable, are difficult to deploy, consume a lot of power, or are difficult to set up. An investigation is required to determine an optimal trade-off on system performance metrics in order to develop an efficient system.

## Figures and Tables

**Table 1:** Position dependent signal parameter comparison.

PDSP	Localization Method	Wireless Technology	Advantages	Disadvantages
TOF	Lateration	Wi-Fi UWB	High localization accuracy	Time synchronization and LOS are required.
AOA	Angulation	Wi-Fi	Require fewer receivers; no time synchronization is necessary	LOS is required, and a smart antenna is needed.
RSS	Fingerprinting Lateration	Wi-Fi Bluetooth Zigbee UWB	Simple and affordable; there is no need for time synchronization; is compatible with a variety of wireless technologies	Fluctuation prone and affected by multipath propagation
CSI	Fingerprinting	Wi-Fi	High accuracy; no need for time synchronization; robust to indoor noise and multipath	Increasingly computationally complex as database size gets bigger; acquirable only for Wi-Fi signal; and very expensive due to special hardware requirements
RTT	Fingerprinting Lateration	Wi-Fi Bluetooth Zigbee UWB	High accuracy; no need for time synchronization; robust to indoor noise and multipath	Limited support from device manufacturer and accuracy depends on clock resolution

**Table 2:** Acronyms and abbreviations.

ANN	Artificial Neural Network
AP	Access point
BLE	Bluetooth
CC	Computational complexity
CNN	Convolution neural network
CSI	Channel state information
DBN	Deep belief network
DNN	Deep neural networks
DOA	Direction of arrival
GMM	Gaussian mixed model
GPS	Global positioning system
GSM	Global System for Mobile Communications
HSPA	High Speed Packet Access
I-WLS	Indoor wireless localization system
k-NN	K-nearest neighbor
LOS	Line-of-sight
LTE	Long-term evolution
ML	Machine learning
NLOS	Non-line-of-sight
PDSP	Position-dependent signal parameter
RF	Reference location
RNN	Recurrent neural network
RSS	Receive signal strength
RSSI	Receive signal strength indicator
SLAM	Simultaneous localization and mapping
SSD	Signal strength difference
SVD	Singular value decomposition
SVM	Support vector machine
TOF	Time of flight
TSVD	Truncated singular value decomposition

**Table 3:** A comparison of existing surveys on fingerprinting-based I-WLS.

Existing Survey	Remark
[5]	Overview of the different I-WLSs and their comparison based on cost and localization accuracy.
[8]	Discusses issues only related to RSS measurement acquisition and the localization ML algorithm.
[21]	Overview of smartphone sensors such as the accelerometer, barometer, and light sensor and how they can be used with various indoor localization techniques.
[18]	Overview of smartphone-based technologies for I-WLS and analysis of different performance metrics and their trade-offs.
[22]	Compares different I-WLS based on energy efficiency, availability, cost, reception range, and latency.
[23]	Comprehensive discussion of the various localization ML techniques used for I-WSL.
[4]	Discussed fingerprinting-based machine learning techniques used in indoor localization.
[25]	Discussed on practical deployment challenges of Wi-Fi-based I-WLSs.
[15]	Discussed on issue related to Wi-Fi-based fingerprinting localization. Issues discussed are radio map construction, outlier detection, and device heterogeneity.
[6]	Discussions on the stages of the Bluetooth-based fingerprint localization system and the classification of the methods used in the different stages.
[16]	Gave an overview of different deep learning methods for fingerprint-based I-WLS.
Current survey	Overview of the different localization performance limiting factors of an RSS fingerprinting-based I-WLS, their effects and possible mitigation techniques.

**Table 4:** Comparison of RSS-based wireless technology.

Wireless Technology	Localization Accuracy	Power Consumption	Maximum Range	Advantages	Disadvantages
Wi-Fi	1 m–5 m	Moderate	10 m–100 m	Complex hardware is not necessary and is widely accessible	Needs a complex processing algorithm
Bluetooth	1 m–4 m	Low	10 m–50 m	Low power requirement, and high throughput	Prone to interference
Zigbee	3 m–5 m	Very low	10 m–100 m	Low cost and energy-efficient	Require additional hardware
UWB	0.1 m–0.5 m	Moderate	4 m–20 m	Immune to interference	Costly, limited in range, and requiring additional hardware

**Table 5:** Summary of techniques on dealing with RSS fluctuation.

Research Work	Summary of Technique
Koubâa et al., (2012) [54]	At each reference location, collect several RSS observations and use a smoothing technique to find a mean RSS representative.
Cheng et al., (2013) [56]	The RSS ratio coefficient, which was obtained using a 3-antenna receiver system, was used instead of measuring aggregated RSS. The log-normal shadowing model is used in the computation of the RSS ratio coefficient.
Ibrahim et al., (2018) [55]	Following the generation of the time-series RSS matrix (concatenation of multiple observations of the RSS fingerprint), the RSS measurements were normalized using the Z-score.
Huang et al., (2019) [57]	Used each BLE advertisement channel instead of the aggregated RSS obtained by the smartphone device.
Polak et al., (2021) [58]	Used a wireless AP with multiple advertising antennas and mean averaging of two consecutives.
Zhou et al., (2021) [61]	Transformed RSS to Q-based RSS and used a Q-based RSS subtraction instead of the direct RSS subtraction.
Balaji et al., (2021) [60]	N RSS measurements were taken for each AP and then arranged in descending order. The median of RSS measurements above the −90 dBm threshold is determined.
Zhou et al., (2022) [59]	Constructed a non-linear and non-stationary RSS sequence, which is then smoothed using an empirical mode decomposition threshold smoothing (EMDT) method.
Flueratoru et al., (2022) [40]	Aggregated RSS measurement from several observations.
Wenqing et al., (2022) [29]	Aggregated 10 RSS measurement obtained by using 10 different parameters in the power-distance equation.
Nabati et al., (2023) [48]	Multiple RSS measurements from each AP at each reference location were averaged.

**Table 6:** Alternative methods for creating radio maps and their drawbacks.

Technique	Limitation
Path loss propagation model	▪ AP locations are required. ▪ Inability to build a precise model due to multipath effects, shadowing, and delay distortions. ▪ Poor performance for a complex indoor environment.
Radio map interpolation	▪ Performance degrades with a large sampling interval and a large number of APs. ▪ Inability to build a precise model due to multipath effects, shadowing, and delay distortions.
Simultaneous localization and mapping	▪ Computationally intensive for smartphones with minimal resources.
Active crowdsourcing fingerprinting	▪ Intentional fraud by participants ▪ Highly sensitive to variations in the devices capturing the measurements. ▪ Prone to fingerprint blind spots. ▪ Multiple users’ multiplication of fingerprints from the same location label wastes storage space on the radio map server.
Passive crowdsourcing fingerprinting	▪ Delays in map construction due to the unavailability of the GPS signal. ▪ Limited applicability to hand-device implementation constraints. ▪ Low accuracy. ▪ Accumulation of IMS errors

**Table 7:** Summary of research on various methods for radio map generation.

Research Works	Summary and Remarks
Khoo et al., (2022) [65]	Based on the k-mean clustering algorithm and dimensional reduction using principal component analysis (PCA) and truncated SVD (TSVD) with an inverse distance weighing interpolation technique.
Assayag et al., (2021) [76]	Based on a weighted average fusion of simulated radio maps generated using the log-distance propagation model by varying different propagation parameters.
Wilson et al., (2021) [77]	Based on a ray-tracing wireless propagation model.
Kolakowski (2020) [67]	Based on active crowdsourcing for initial radio map generation and the use of interpolation technique to add missing fingerprints.
Kolakowski (2021) [66]	Based on LiDAR Graph-SLAM-based algorithm for environmental mapping and interpolation technique.
Kawecki et al., (2022) [68]	Based on raytracing and multiwall propagation models.

**Table 8:** Advantages and disadvantages of commonly used fingerprinting-based ML algorithms.

ML Algorithm	Advantages	Disadvantages
k-NN	Simple to compute; less time for parameter optimization	Finding the right k value is challenging; covers only the region contained within the convex hull of the reference locations; does not perform well with large fingerprint datasets; susceptible to noisy and missing fingerprints; poor label selection accuracy
W*k*-NN	Improved label selection accuracy; preferred choice for sizable mixed environments	Finding the right k value is challenging; covers only the region contained within the convex hull of the reference locations; does not perform well with large fingerprint datasets; susceptible to noisy and missing fingerprints
Adaptive W*k*-NN	Dynamic selection of the k value; enhanced label selection precision	Covers only the region contained within the convex hull of the reference locations; does not perform well with large fingerprint datasets; susceptible to noisy and missing fingerprints
GMM	Very fast localization time; robust to noise in the dataset	Creating a good representative probability density function for fingerprints requires greater samplings
SVM	Fast convergence rate; training and computation time reduction in small environments	Incorrect parameter selection has an impact on its performance
Random Forest	Work incredibly well with a huge fingerprint dataset; reduce redundant measurements	Very sensitive to parameter changes and computationally demanding for large fingerprint datasets
Deep learning	Robustness against noise and interference; works well in a dynamic environment	Requires significant computational resources and storage for complex feature extraction

**Table 9:** Computational complexity comparison.

ML Algorithm	Complexity	Symbol and Notations
k-NN	$O\left(mn\right)$	*m* is the number of features *n* is the number of training samples
GMM	$O\left(mn_{GC}d\right)$	*m* is the number of features $n_{GC}$ is the number of Gaussian components *d* dimension of problem
SVM	$O\left(mn_{sv}\right)$	*m* is the number of features $n_{sv}$ is the number of support vectors
Random Forest	$O\left(mn_{tree}\right)$	$n_{tree}$ is the number of trees *m* is the number of features
Decision Tree	$O\left(m\right)$	*m* is number of features
Neural Network	$O\left(mn_{1}+n_{l_{1}}n_{l_{2}}+\ldots \right)$	*m* is the number of features $n_{l_{i}}$ is the number of neurons at the *i*-th layer in a neural network

**Table 10:** Characteristic comparison of online RSS Fingerprint datasets.

Year	Dataset	Technology	Coverage Area $\left(m^{2}\right)$	No. of Floors	No. of AP	No. of Devices	No. of Samples	No. of Reference Locations
2014	[91]	Wi-Fi	108,703	4	520	25	21,049	933
2018	[95]	Wi-Fi	308	2	448	1	63,504	212
2018	[94]	BLE	388.2	2	11	1	73,000	735,417
2018	[93]	BLE	237.38	1	185	1	2,820,000	277
2022	[41]	Cellular	1	1	1	1	3,408	32
2022	[92]	Wi-Fi	8000	3	105	1	23,925	1802

## Data Availability

Not applicable.
